# Primary leiomyosarcoma of a horseshoe kidney in a woman with Turner syndrome: a case report

**DOI:** 10.1186/1756-0500-7-491

**Published:** 2014-08-04

**Authors:** Toshikazu Tanaka, Takuya Koie, Ikuya Iwabuchi, Masaru Ogasawara, Toshiaki Kawaguchi, Chikara Ohyama

**Affiliations:** 1 Department of Urology, Aomori Prefectural Central Hospital, 2-1-1 Tsukurimichi, Aomori 030-8553, Japan; 2 Department of Urology, Hirosaki University Graduate School of Medicine, 5 Zaifucho, Hirosaki 036-8562, Japan

**Keywords:** Leiomyosarcoma, Turner syndrome, Horseshoe kidney

## Abstract

**Background:**

Turner syndrome is characterized by complete or partial X-chromosome monosomy and has various clinical features, including horseshoe kidney. Leiomyosarcoma is an extremely rare tumor that accounts for only 0.1% of all invasive renal tumors.

**Case presentation:**

A 50-year-old Japanese woman presented at a community hospital with a chief complaint of abdominal pain. Computed tomography revealed a horseshoe kidney with a hypovascular tumor (size, 9 × 7 cm) showing calcification in the upper pole of the right kidney. Open right heminephrectomy and division of the isthmus were performed. Histological examination revealed alternating fascicles of spindle cells with blunt ended non-tapering nuclei and eosinophilic cytoplasm. The tumor had high mitotic activity with a mitotic count of 8 mitoses/10 high-power fields. On the basis of these findings, we diagnosed the patient as having leiomyosarcoma.

**Conclusion:**

Primary leiomyosarcoma of the horseshoe kidney in a patient with Turner syndrome is a very rare occurrence.

## Background

Turner syndrome (TS) affects approximately 1 in 2,000 live-born girls [1]. This condition is characterized by complete or partial X-chromosome monosomy. The typical clinical features are short stature, ovarian dysgenesis with concomitant primary amenorrhea, and lymphedema. Horseshoe kidney has been observed in approximately 30% of patients with TS [2]. Although primary leiomyosarcoma is the most common de novo renal sarcoma of the kidney, it is an extremely rare entity that accounts for only 0.1% of all invasive renal tumors [3]. Here, we report a unique case of leiomyosarcoma arising in the horseshoe kidney of a patient with TS.

## Case presentation

**Figure 1:**
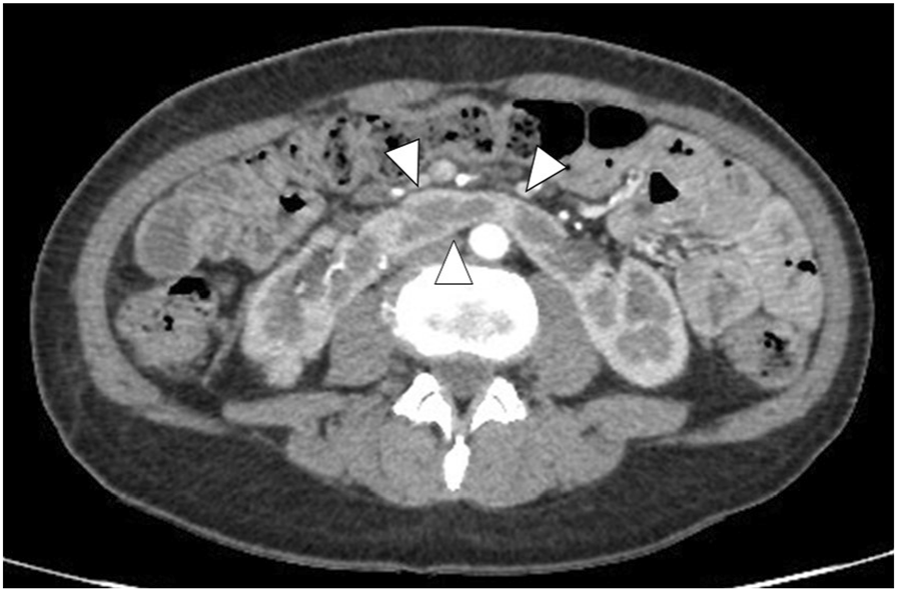
**Enhanced abdominal computed tomography.** Abdominal computed tomography revealed a horseshoe kidney. The arrowheads indicated the isthmus of the horseshoe kidney.

**Figure 2:**
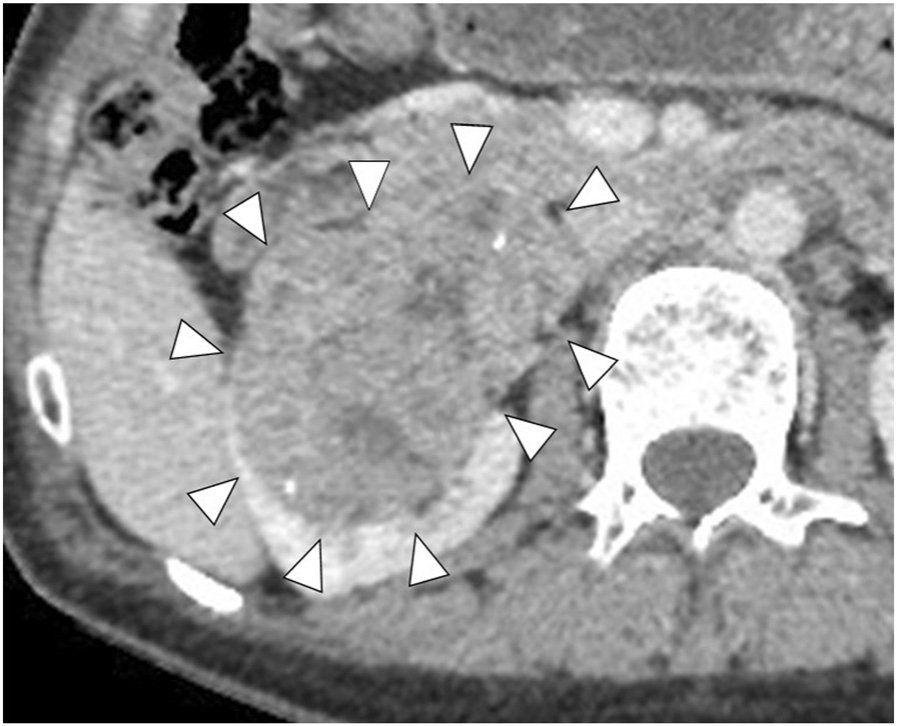
**Enhanced abdominal computed tomography.** Abdominal computed tomography revealed a hypovascular tumor, measuring 9 × 7 cm, in the upper pole of the right kidney (arrowheads).

**Figure 3:**
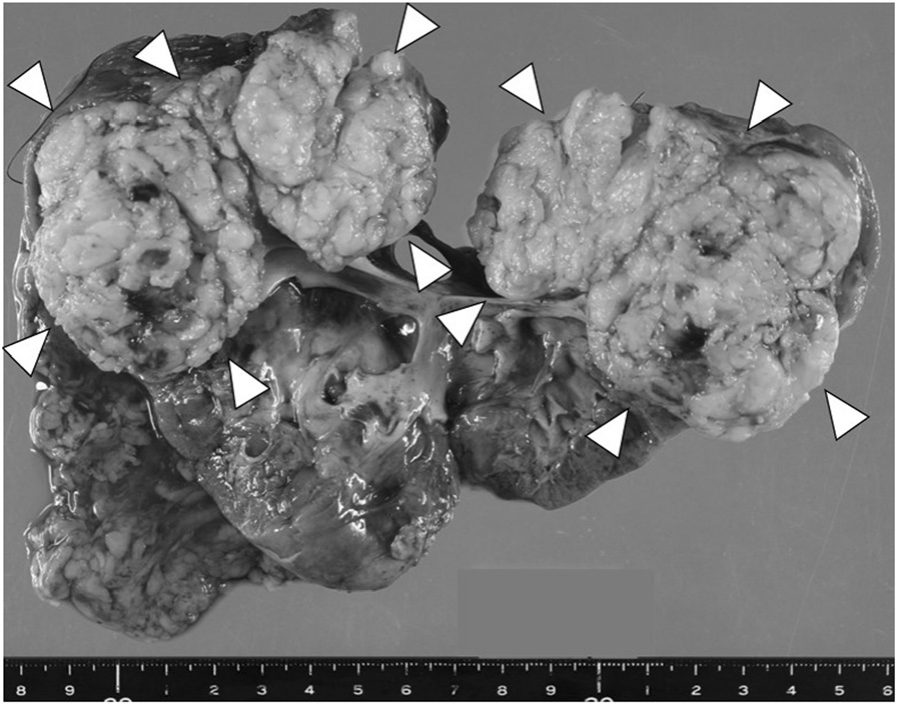
**Macroscopic findings.** Macroscopic examination revealed a solid, circumscribed, and yellowish-white tumor, measuring 9 × 7 cm in size, in the upper pole of the resected kidney (arrowheads).

**Figure 4:**
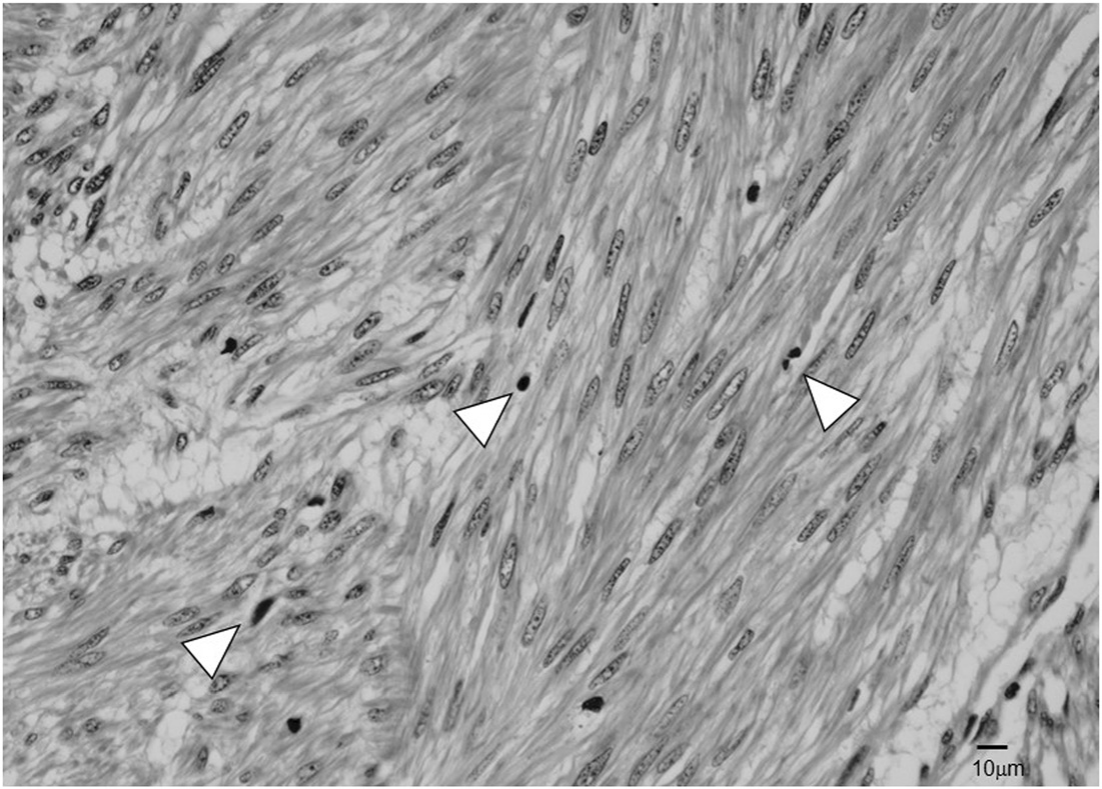
**Hematoxylin-eosin stained section of the tumor.** The cells had alternating fascicles of spindle cells with blunt ended non-tapering nuclei and eosinophilic cytoplasm (arrowheads) (magnification, 200×).

**Figure 5:**
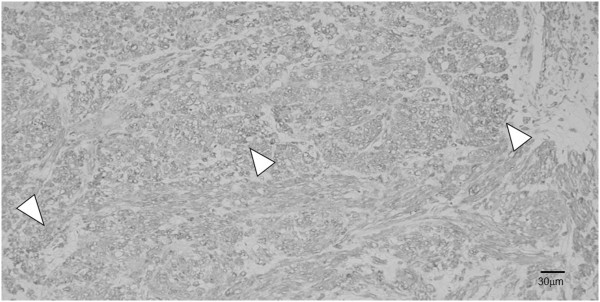
**Section of the tumor stained for alpha-smooth muscle actin.** The tumor cells were strongly positive for alpha-smooth muscle actin (arrowheads) (magnification, 100×).

**Figure 6:**
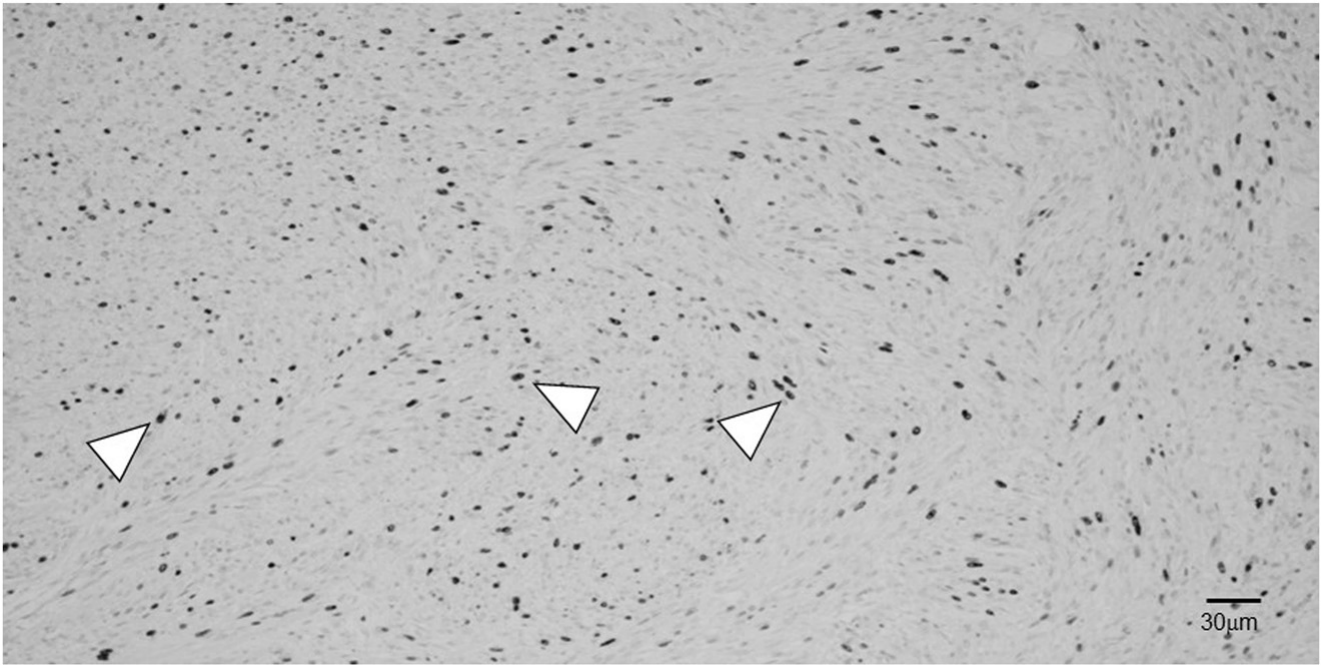
**Section of the tumor stained for Ki-67.** The tumor cells were strongly positive for Ki-67 (arrowheads) (magnification, 100×).

A 50-year-old Japanese woman presented at a community hospital with a chief complaint of abdominal pain. Computed tomography revealed a horseshoe kidney (Figure 1) with a hypovascular tumor (size, 9 × 7 cm) showing calcification in the upper pole of the right kidney (Figure 2). The tumor was clinically diagnosed as a right renal cell carcinoma (RCC) and was classified as cT3aN0M0, according to the tumor-node-metastasis system [4]. Open right heminephrectomy and division of the isthmus were performed. Macroscopic examination revealed a solid, circumscribed, and yellowish-white tumor (size, 9 × 7 cm) in the upper pole of the resected kidney (Figure 3). Histological examination revealed alternating fascicles of spindle cells with blunt ended non-tapering nuclei and eosinophilic cytoplasm (Figure 4). The tumor had high mitotic activity with a mitotic count of 8 mitoses/10 high-power fields. Immunohistochemical analysis revealed that the tumor cells were strongly positive for alpha-smooth muscle actin (Figure 5), desmin, vimentin, and Ki-67 (Figure 6). On the basis of these findings, we diagnosed the patient as having stage pT2aN0M0 leiomyosarcoma.

The patient was asymptomatic and disease free at 6 months after the diagnosis.

## Conclusions

To the best of our knowledge, this is the first reported case of primary leiomyosarcoma arising in a horseshoe kidney in a patient with TS.

The risk of cancer in patients with TS has been established. The overall risk of cancer is similar to that in the general population [5]. However, in patients with TS, site-specific risks were significantly increased for meningeal tumors, childhood brain tumors, bladder and urethral cancer, and ocular cancer and significantly decreased for breast cancer [5]. Therefore, the hormonal abnormalities associated with TS might affect the risk of hormone-related cancers, and the chromosomal abnormality itself might affect the cancer risk. The cancer risk was potentially related to the absence of one or more genes on the X chromosome that escape X-inactivation [6]. The X chromosome has many cancer-related genes [7], and allelic losses from the X chromosome have been noted in several malignancies [8,9]. Such losses suggest that the presence of one or more tumor-suppressor or DNA-repair genes on the X chromosome is relevant to the etiology of these tumors [5].

The phenotype of TS is highly variable, with a wide variety of clinical conditions. Renal and/or collecting system malformations, including horseshoe kidney, renal malrotation, and collecting system malformations, have been observed in 30–40% of patients with TS [2]. Although most structural anomalies may initially be asymptomatic, there may be a high risk of hypertension, urinary tract infection, and hydronephrosis [2]. The incidence of tumors in a horseshoe kidney is approximately 3–4 times higher in patients with TS than in the general population [10]. Therefore, it has been suggested to be caused by chronic obstruction, lithiasis, and infection. RCC is the most common neoplasm associated with horseshoe kidney and is mostly found adjacent to or within the isthmus [11]. Furthermore, horseshoe kidney is associated with a 2-fold increase in the relative risk of Wilms tumor and a 3- to 4-fold increase in the risk of transitional cell carcinoma [12].

Primary renal sarcomas are rare in adults and account for approximately 1% of all primary renal malignancies, whereas leiomyosarcoma constitute approximately 50–60% of all cases of renal sarcoma [13]. Leiomyosarcoma of the kidney most likely arises from the renal capsule, smooth muscle fibers of the renal pelvis, sphincter ring around the renal papilla, and internal blood vessels [14]. In contrast, the vast majority of malignant spindle cell tumors of the kidney represent a component of a sarcomatoid carcinoma [13]. Sarcomatoid carcinomas arise from either renal cell carcinoma or, less commonly, urothelial carcinomas of the renal pelvis [15]. Although the spindle cell component consisting of sarcomatoid RCC is often positive for epithelial markers, leiomyosarcoma is very rarely positive for keratins [16].

A few large series on primary leiomyosarcoma of the kidney with an analysis of the prognosis have been reported. Kendal analyzed the population-based surveillance, epidemiology, and end results (SEER) registry and reported on the survival of 112 patients with renal leiomyosarcomas [3]. On the basis of the SEER data, the median overall survival (OS) was 25 months and the 5-year OS rate was 25%. The tumor stage and age at diagnosis were independent predictive factors for OS [3].

In conclusion, primary leiomyosarcoma of the horseshoe kidney in a patient with TS is a very rare occurrence. It is important to remember that this unusual variant of renal leiomyosarcoma has the potential for aggressive behavior, which can lead to metastasis.

## Consent

Written informed consent was obtained from the patient for publication of this Case Report and any accompanying images. A copy of the written consent is available for review by the Editor-in-Chief of this journal.

## Abbreviations

TS: Turner syndrome; RCC: Renal cell carcinoma; SEER: Surveillance, epidemiology, and end results; OS: Overall survival.

## Competing interests

The authors declare that they have no competing interests.

## Authors’ contributions

YT and TK were involved in drafting the manuscript. TK performed the clinical follow-up and contributed to manuscript preparation. KO and II performed the surgery. TK and CO were responsible for the concept and design of this study, interpretation of the data, and critical revision of the manuscript. All authors read and approved the final manuscript.
